# Characterization of temperature and light effects on the defense response phenotypes associated with the maize *Rp1-D21* autoactive resistance gene

**DOI:** 10.1186/1471-2229-13-106

**Published:** 2013-07-26

**Authors:** Adisu Negeri, Guan-Feng Wang, Larissa Benavente, Cromwell M Kibiti, Vijay Chaikam, Guri Johal, Peter Balint-Kurti

**Affiliations:** 1 Department of Plant Pathology, North Carolina State University, Raleigh, NC 27695, USA; 2 USDA-ARS, Plant Science Research Unit, North Carolina State University, Raleigh, NC 27695, USA; 3 Department of Botany and Plant Pathology, Purdue University, West Lafayette, IN 47907, USA

**Keywords:** Maize, Hypersensitive response, Disease resistance, Temperature sensitive, Light dependent, *Rp1*, Autoactive R gene

## Abstract

**Background:**

*Rp1* is a complex locus of maize, which carries a set of genes controlling race-specific resistance to the common rust fungus, *Puccinia sorghi*. The resistance response includes the “Hypersensitive response” (HR), a rapid response triggered by a pathogen recognition event that includes localized cell death at the point of pathogen penetration and the induction of pathogenesis associated genes. The *Rp1-D21*gene is an autoactive allelic variant at the *Rp1* locus, causing spontaneous activation of the HR response, in the absence of pathogenesis. Previously we have shown that the severity of the phenotype conferred by *Rp1-D21* is highly dependent on genetic background.

**Results:**

In this study we show that the phenotype conferred by *Rp1-D21* is highly dependent on temperature, with lower temperatures favoring the expression of the HR lesion phenotype. This temperature effect was observed in all the 14 genetic backgrounds tested. Significant interactions between the temperature effects and genetic background were observed. When plants were grown at temperatures above 30°C, the spontaneous HR phenotype conferred by *Rp1-D21* was entirely suppressed. Furthermore, this phenotype could be restored or suppressed by alternately reducing and increasing the temperature appropriately. Light was also required for the expression of this phenotype. By examining the expression of genes associated with the defense response we showed that, at temperatures above 30°C, the *Rp1-D21* phenotype was suppressed at both the phenotypic and molecular level.

**Conclusions:**

We have shown that the lesion phenotype conferred by maize autoactive resistance gene *Rp1-D21* is temperature sensitive in a reversible manner, that the temperature-sensitivity phenotype interacts with genetic background and that the phenotype is light sensitive. This is the first detailed demonstration of this phenomenon in monocots and also the first demonstration of the interaction of this effect with genetic background. The use of temperature shifts to induce a massive and synchronous HR in plants carrying the *Rp1-D21* genes will be valuable in identifying components of the defense response pathway.

## Background

The *Rp1* locus of maize carries variable numbers of tandemly-repeated genes belonging to the nucleotide binding site, leucine-rich repeat (NB-LRR) class of major disease resistance genes (also known as *R*-genes; [1]) that confer race-specific resistance to the common rust fungus, *Puccinia sorghi*[2]. The resistance response conferred by these genes is generally activated by a race-specific direct or indirect recognition of pathogen-derived molecules [1]. It includes the “hypersensitive response” (HR) which is a rapid localized cell death at the point of pathogen penetration, the induction of pathogenesis associated genes and a variety of other responses [3].

The *Rp1* locus is complex; the well characterized *HRp1-D* haplotype is composed of nine tandemly-repeated *Rp1* paralogs [4] while the *Rp1* haplotype of the B73 line carries four *Rp1*-homologous sequences [5] and more than 50 *Rp1*-homologous sequences have been identified in other lines [6]. This complexity results in the locus being meiotically unstable [7,8], with a high frequency of unequal crossover events [9]. Intragenic recombination events resulting in the generation of chimeric genes derived from two different *Rp1* paralogs have been observed several times. In some cases these lead to the generation of novel resistance specificities [10]. In other cases it has led to the creation of dominant mutant genes that confer a “spontaneous necrotic” or “lesion” phenotype [10-13].

*Rp1-D21* is one such gene [13]. It confers a spontaneous lesion phenotype with lesions forming in apparently random locations without the requirement for the presence of a pathogen. Previously we showed that the *Rp1-D21* phenotype is influenced by genetic background; in some backgrounds it is very severe, killing the plant during the juvenile phase. In others it is suppressed to the extent that the mutant plants can set seed in a more-or-less normal fashion [3,14,15].

The signal transduction pathway leading to the HR response and the mechanism by which the plant is able to restrict the HR to cells in the immediate area surrounding an infection is poorly understood in plants. We are using a series of genetic crosses with diverse lines and with members of segregating populations to identify loci and genes that interact with *Rp1-D21* either to suppress or enhance its expression. The expectation is that many of these loci are also important in the regulation of the ‘normal’ HR induced by wild type *R*-genes in response to pathogen detection [3,14,15]. This approach, in which a mutant conferring an extreme phenotype in a trait of interest is used to uncover previously inaccessible genotype-dependent variation has been termed Mutant-Assisted Gene Identification and Characterization (MAGIC) [16,17].

Temperature has long been known to play a role in the modulation of plant defense responses in dicots [18], including the HR [19-22]. In some cases it appears that the temperature-sensitive component of the defense response resides in the NB-LRR genes themselves [23]. Light sensitivity of resistance responses, including HR, is likewise a well known phenomenon [24,25]. In the course of our studies we noted that phenotypic expression of *Rp1-D21* seemed to be affected by temperature. This effect had been briefly noted previously [11]. In light of our ongoing work on the genetic dissection of HR using *Rp1-D21* in a MAGIC approach, and especially because these experiments were taking place in multiple locations under varying environmental conditions, it was important to characterize this temperature-dependent effect further and to ascertain whether there was an interaction with genotype. We show here that while temperature effects on the *Rp1-D21* phenotype were observed in all 14 genetic backgrounds tested, the temperature effects were significantly affected by genotype. The phenotype could be completely suppressed in a reversible way by manipulating temperature or light levels.

## Results and discussion

### *Rp1-D21*-associated lesion phenotypes are temperature dependent

The *Rp1-D21*-H95 line has the background of the common maize inbred H95 into which the *Rp1-D21* allele has been introgressed. It is 97% genetically identical to H95 [14]. *Rp1-D21*-H95 plants are heterozygous for the *Rp1-D21* mutant allele and therefore the F_1_ progeny of any cross between *Rp1-D21*-H95 and another line segregate in a 1:1 ratio for F_1_ progeny carrying an *Rp1-D21* gene (i.e. mutant F_1_ progeny) and F_1_ progeny carrying the wild-type (i.e. non-autoactive) H95 allele at the *Rp1* locus (i.e. wild type F_1_ progeny). Importantly the wild type and mutant F_1_ progeny were almost entirely isogenic outside the *Rp1* locus [14].

The *Rp1-D21*-H95 line and crosses between the *Rp1-D21*-H95 line and 13 diverse lines were used for this study (Table 1). All of these lines are among the 26 parents of the maize nested association mapping (NAM) population [26]. Importantly for these purposes, these lines represent a diversity of phenotypic effects on the *Rp1-D21* phenotype. Table 2 is adapted from Chintamanai et al. [3] and shows the response of these crosses in the field in two environments, Clayton NC and West Lafayette IN, that we observed in our previous study. Included in this experiment are crosses in which the *Rp1-D21* phenotype is substantially suppressed (B97 × *Rp1-D21*-H95, Mo18W × *Rp1-D21*-H95, Oh7B × *Rp1-D21*-H95) and several in which it is substantially enhanced (Tx303 × *Rp1-D21*-H95, M37W x *Rp1-D21*-H95, M162W × *Rp1-D21*-H95 ) as well as several crosses with intermediate phenotypes.

**Table 1:** **Mean lesion score (LES), and mean mutant to wild type height ratio (HTR) for crosses of various lines to *Rp1-D21*-H95 grown under three different greenhouse temperature regimes**

		**HiGH (30/26°C)**		**MidGH (26/22°C)**		**LowGH (22/18°C)**	
**Germplasm**	Group*	**LES**	**HTR**	**LES**	**HTR**	**LES**	**HTR**
B97 × *Rp1-D21*-H95	NSS	2.00	0.89	5.08	0.76	5.83	0.87
CML103 × *Rp1-D21*-H95	TS	3.88	0.89	6.88	0.58	6.69	0.55
CML228 × *Rp1-D21*-H95	TS	2.75	0.89	5.00	0.79	5.13	0.67
CML277 × *Rp1-D21*-H95	TS	3.88	0.86	7.00	0.61	6.60	0.65
CML322 × *Rp1-D21*-H95	TS	5.63	0.80	9.00	0.50	7.98	0.38
Ky21 × *Rp1-D21*-H95	NSS	5.67	0.81	8.67	0.34	8.38	0.33
M162W × *Rp1-D21*-H95	NSS	8.25	0.74	9.17	0.38	9.50	0.33
M37W × *Rp1-D21*-H95	Mixed	6.31	0.76	8.67	0.45	8.63	0.31
Mo18W × *Rp1-D21*-H95	Mixed	4.00	0.98	4.88	0.87	6.08	0.31
MS-71 × *Rp1-D21*-H95	NSS	3.88	0.76	6.88	0.57	-	-
OH43 × *Rp1-D21*-H95	NSS	2.00	0.96	-	-	4.00	-
OH7B × *Rp1-D21*-H95	Mixed	2.00	0.93	5.13	0.91	6.25	0.84
Tx303 × *Rp1-D21*-H95	Mixed	7.00	0.77	8.13	0.35	9.67	0.25
*Rp1-D21*-H95	NSS	3.00	0.91	4.00	0.66	-	-

*Major breeding group to which the female parent belongs as defined in Liu et al. (2003), Table 2.

Data are derived from experiments conducted during fall 2009 and fall 2010. In each year the experiment was laid out in randomized complete block design with two replicates per year per temperature condition. LES was scored on a 0–10 scale with 0 being no lesions at all and 10 being dead. Missing data are denoted by a dash (-).

**Table 2:** **The diversity of the *Rp1-D21 *mediated HR in maize [Adapted from Table**1**in reference 3]**

	**Indiana scores**		**NC scores**	
**Cross**	**HTR**	**LES**	**HTR**	**LES**
B97 × *Rp1-D21*-H95	0.82	2	0.73	3.40
A632 × *Rp1-D21*-H95	0.8	1.5	0.67	2.3
Oh43 × *Rp1-D21*-H95	0.76	3.5	0.89	2.74
CML228 × *Rp1-D21*-H95	0.73	3.5	0.89	1.92
Mo18w × *Rp1-D21*-H95	0.68	3	0.66	4.28
Oh7B × *Rp1-D21*-H95	0.68	3	-	-
B73 × *Rp1-D21*-H95	0.64	4	0.74	2.2
CML333 × *Rp1-D21*-H95	0.4	4	0.69	3.11
Mo17 × *Rp1-D21*-H95	0.39	6	0.48	6.3
MS-71 × *Rp1-D21*-H95	0.35	6.5	0.82	5.22
CM103 × *Rp1-D21*-H95	0.23	7	0.50	5.61
CML277 × *Rp1-D21*-H95	0.19	7	0.62	3.64
Ky21 × *Rp1-D21*-H95	0.18	9	0.37	6.56
M162W × *Rp1-D21*-H95	0	10	0.30	7.84
M37W × *Rp1-D21*-H95	0	10	0	10
Tx303 × *Rp1-D21*-H95	0	10	0.34	6.33

F_1_ families were derived from crosses between a number of inbreds and the *Rp1-D21*-H95 line, which was heterozygous for the *Rp1-D21* gene. The resulting F_1_ families therefore segregated 1:1 for the necrotic spotting phenotype associated with *Rp1-D21*. Measured in Clayton NC in 2006 and in West Lafayette IN in 2009, lesion score (LES) and mutant to wild type height ratio (HTR) were measured as described in the materials and methods.

**Figure 1:**
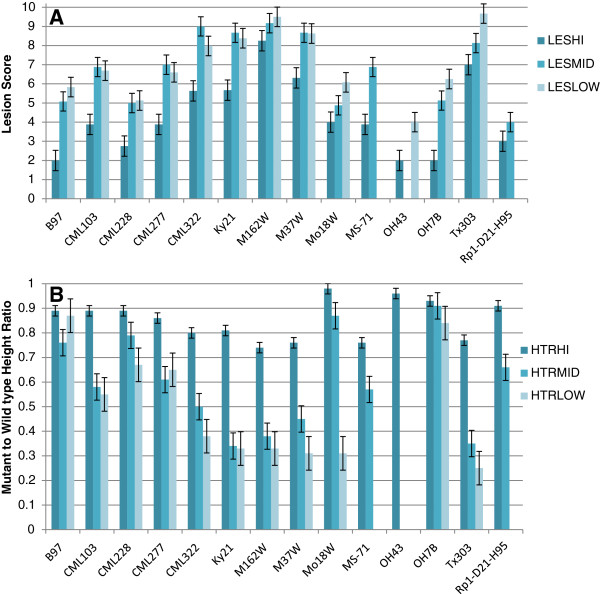
**Phenotype of *Rp1-D21 *in 14 different genetic backgrounds at three different temperatures. (A)** Mean lesion score (LES), and **(B)** mean mutant to wild type height ratio (HTR) for crosses of various lines to *Rp1-D21*-H95 grown under three different greenhouse temperature regimes, 22/18°C (LOW), 26/22°C (MID), and 30/26°C (HI). Data are derived from experiments conducted during fall 2009 and 2010. LES was scored on a 0–10 scale with 0 being no lesions at all and 10 being dead.

These F_1_ crosses, together with the *Rp1-D21*-H95 line were grown in the greenhouse at three different temperature regimes: 22/18°C, 26/22°C, and 30/26°C higher/ lower for 12 hours each. For simplicity, these regimes are referred to here as, respectively, LowGH, MidGH, and HiGH (see Methods). Two traits associated with the severity of the *Rp1-D21* phenotype were scored on all the plants, mean lesion score (LES), and mean mutant to wild type height ratio (HTR). LES is simply a score of the lesion severity (Additional file 1: Figure S1) while HTR determines how much the growth of the plant has been inhibited by the expression of *Rp1-D21* with respect to a near-isogenic sibling lacking the *Rp1-D21* gene. The mean LES and HTR scores for all crosses at each temperature are summarized in Table 1. The plants grown at the highest temperatures, 30/26°C, had the most suppressed *Rp1-D21* phenotypes (lowest LES score, highest HTR) in every case regardless of genotype. However, the ranking of these two traits at 18/22°C and 22/26°C depended on the genetic background although in general the phenotype was most extreme at the lower temperature (Figure 1). The results observed in this study are largely consistent with our previous work [3] with the Tx303 × *Rp1-D21*-H95, M37W × *Rp1-D21*-H95, M162W × *Rp1-D21*-H95 F_1_ crosses showing the most severe phenotypes and the B97 × *Rp1-D21*-H95, Mo18w × *Rp1-D21*-H95, Oh7B × *Rp1-D21*-H95 F_1_ crosses among the most suppressed.

Analysis of variance (ANOVA) showed that the most significant sources of variation for both LES and HTR were environment (meaning the two different occasions on which the experiment was performed in 2009 and 2010), temperature and genotype. There were also highly significant (*p* ≤ 0.001) interactions between genotype and environment, and genotype and temperature for both traits (Table 3).

**Table 3:** **ANOVA for lesion score (LES) and mutant to wild type height ratio (HTR) for 13 F**_**1 **_**families derived diversity maize lines crossed to *Rp1-D21*-H95 grown at three temperature ranges in two experiments during fall 2009 and fall 2010**

**Source of variance**	**DF**	**MSS**	
		**LES**	**HTR**
Genotype	13	23.09**	0.17**
Environment	1	93.56**	0.40**
Temperature	2	54.61**	0.89**
Replicate(Environment)	2	1.16**	0.01ns
Maize founder lines*Environment	11	2.68**	0.02ns
Maize founder lines *temperature	23	0.89**	0.02**
Environment*Temperature	2	3.71**	0.08**
Maize founder lines *temperature*Environment	16	1.47**	0.03**
Error	52	0.19	0.01

**, Significant at probability level, *p* ≤ 0.01, ns=not significant, DF=Degree of freedom, MSS= mean sum of squares.

LES was scored on a 1–10 scale with 1 being no lesions at all and 10 being dead.

Since LES and HTR measure aspects of the same phenotype, it was not surprising that they were highly correlated. Combined data from Fall 2009 and Fall 2010 showed strong negative phenotypic correlation between LES and HTR (Pearson correlation coefficient = -0.77, *p* < 0.001). At each temperature range the correlation was strong (Pearson correlation coefficient =-0.79, -0.78, and -0.44 at 18/22°C, 22/26°C, and 26/30°C, respectively). The correlation showed a trend of weakening association between LES and HTR as temperature increased. This was probably because higher temperature suppressed the phenotypic expression of the *Rp1-D21* phenotype.

### The effect of temperature reduction on the *Rp1-D21*-associated lesion phenotype

**Figure 2:**
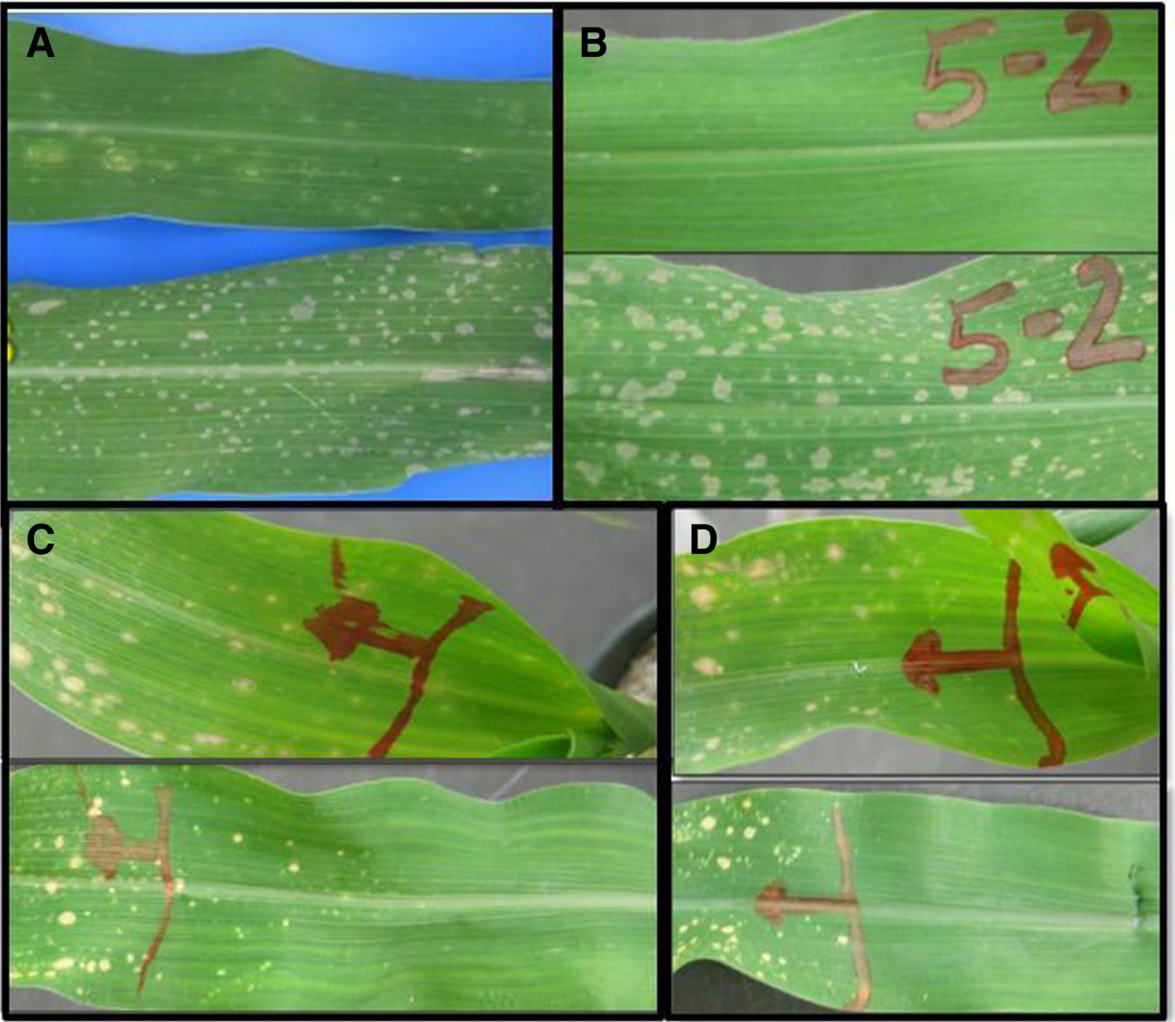
**Effects of temperature shifts on the *Rp1-D21 *phenotype. (A)** Mutant plants from the cross TX303 × *Rp1-D21*-H95 showed a dramatic phenotypic change from minor lesions (upper leaf) to more severe lesions (lower leaf) 48 hours after moving from 30/26°C to 22/18°C. **(B)** Mutant plants from the cross M37W ×*Rp1-D21*-H95 showed no lesions after three weeks growth at 34/30°C (upper picture). The lower picture shows the identical leaf 48 hours after changing the temperature to 22/18°C. **(C** and **D)** The point at which the growing leaf emerged from the main stalk was marked on a leaf of a mutant plants from the cross M37W ×*Rp1-D21*-H95 **(C)** and B97 ×*Rp1-D21*-H95 **(D)** growing at 22/18°C (upper picture). The temperature was then increased to 34/30°C. The lower picture shows the identical leaf seven days later.

To investigate the effect of changing the external temperature on the expression of the *Rp1-D21* phenotype, we moved some Tx303 × *Rp1-D21*-H95 mutant plants from the HiGH to the LowGH. Within 48 hours a dramatic phenotypic change was noticed; leaves which had displayed sparse small lesions developed much more numerous and larger lesions within 48 hours of the transfer to lower temperatures (Figure 2A). Genetically identical plants that remained in HiGH conditions did not display this dramatic change (data not shown).

We subsequently conducted an experiment in which eight F_1_ seed from each of four families from the crosses of *Rp1-D21*-H95 to M37W, Tx303, M162W and B97, which represent a diversity of *Rp1-D21*supressive and enhancing backgrounds, were planted in growth chambers at 30/34°C (12 hours/12 hours)- i.e. 4°C higher than the HiGH. After three weeks growth at 30/34°C (~four leaf stage) no lesions were visible on any of the plants and it was impossible to discern wild type from mutant segregants. In one growth chamber the temperature was then dropped to 18/22°C. After 48 hours, half the plants (i.e. presumably the plants carrying the *Rp1-D21* gene) from the M37W, Tx303, M162W F_1_ families developed copious lesions (an example is shown in Figure 2B). The plants from the B97 F_1_ family took longer to develop lesions but after seven days some lesions were visible on about half of the plants from this family too. In the growth chamber which had remained at 30/34°C all the plants still were indistinguishable from wild type and no lesions were visible on any leaves.

### The HR-phenotype associated with *Rp1-D21* can be activated/inactivated reversibly using temperature shifts

At the four-week time point and using the same plants that had been shifted from 30/34°C to 18/22°C, the location at which the emerging leaves joined the main stem was marked on the mutant plants and the temperature of the growth chamber was increased from 18/22°C back to 30/34°C. After seven days, all the newly-emerged leaves and the newly-emerged portions of the leaves that had been partially emerged at four weeks showed no, or in just a few cases, a very few lesions (Figure 2C, D).

In a separate experiment (Additional file 2: Figure S2) we grew at least 3 mutant F_1_ plants from each of 13 different genotypes for four weeks at 22 /18°C and then shifted to 34/30°C. At this point the 5th leaves were partially emerged from the whorl. In every case a clear transition from a lesioned to a non-lesioned phenotype was apparent on the 5th leaf (Additional file 2: Figure S2).

This result implied two things: Firstly that the *Rp1-D21* lesion phenotype could be turned off, then on, then off again simply by increasing the temperature above about 30°C and decreasing it to about 20°C by turns. In other words the activation/inactivation of the HR phenotype was reversible. Secondly, since no lesions were visible on portions of the plant that emerged immediately after the temperature was increased to 30/34°C, it implied that the *Rp1-D21*-associated lesions did not form before leaf emergence from the whorl but that something associated with leaf emergence, perhaps exposure to light, was required for the lesion phenotype.

### An objectively measurable quantitative assay for *Rp1-D21* temperature-sensitive phenotype

To examine the detailed kinetics of *Rp1-D21* lesion development following transfer to lower temperatures, four different genotypic combinations were selected: F_1_ progenies of *Rp1-D21-*H95 crossed to A632, B73, Mo17 and Tx303. The phenotype underlying *Rp1-D21* is highly suppressed in a hybrid combination of H95 with A632, but highly enhanced in a hybrid combination with Tx303 while F_1_ hybrids of *Rp1-D21-*H95 with B73 and Mo17 exhibit intermediate phenotypes (Table 2).

**Figure 3:**
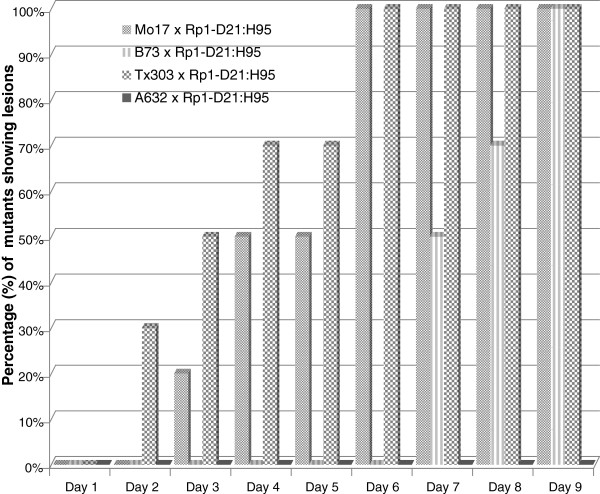
**Percentage (%) mutants expressing *Rp1-D21 *lesions following transfer from 30°C to 26°C.** In each case percentages are calculated from a total of 10 plants observed.

None of the mutants F_1_s developed *Rp1-D21*-associated lesions in any genetic background when grown at 30°C. Eight days after planting, the temperature was lowered to 26°C at which point HR lesions started to become apparent. The speed at which the HR lesions started manifesting was dependent on genotype of the host (Figure 3). The genetic backgrounds that had previously been associated with a more severe *Rp1-D21* phenotype (Table 2), *Rp1-D21-*H95 × Tx303 and *Rp1-D21-*H95 × Mo17, displayed lesions within two and three days respectively. Of the backgrounds associated with more suppressed phenotypes, B73 took seven days for half the plants to display lesions while lesions had not formed on *Rp1-D21-*H95 × A632 mutants at 26°C plants nine days after the temperature shift. These results demonstrate how the temperature-dependent phenotype of *Rp1-D21* can be converted to an objectively measurable quantitative trait. Loci responsible for mediating this temperature sensitivity could therefore by mapped using an approach similar to that which enabled us to map loci associated with *Rp1-D21*-mediated lesion severity in the field [3,14].

### Light requirement for *Rp1-D21* lesion manifestation

**Figure 4:**
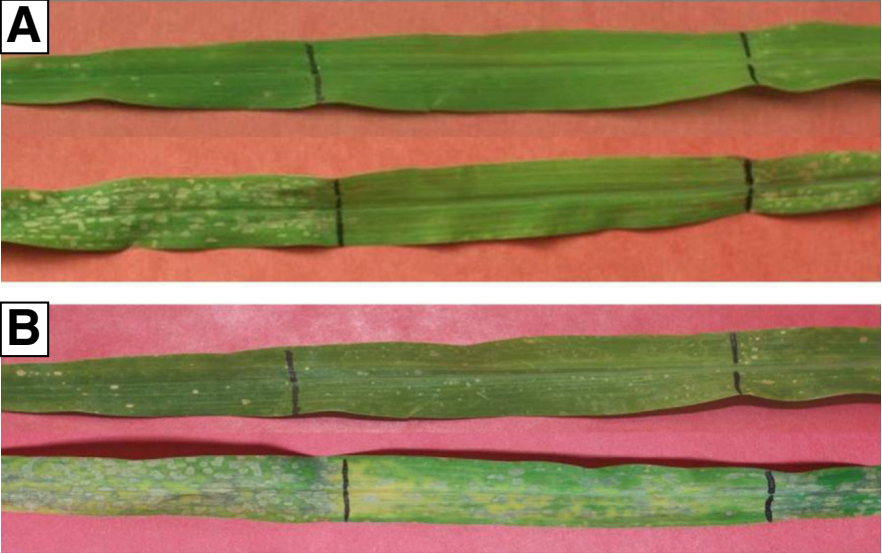
**Seedlings of *Rp1-D21*-H95 (top) and Tx303 × *Rp1-D21*-H95 (bottom) were grown in 34/30°C, with 12 h light/12 h dark for 10 days.** The middle section of the 4th leaves were wrapped in aluminum foil and the temperature was changed to 22/18°C. **(A)** Foil was removed after a further 4 days at the lower temperature. **(B)** Two days after removal of foil still at the lower temperature. Lines drawn on the leaf indicate where the aluminum foil was placed.

Since it appeared that lesions formed only after emergence from the whorl (see above), we wanted to determine whether exposure to light was important for lesion formation. In a separate experiment, eight seedlings of Tx303 × *Rp1-D21*-H95 and *Rp1-D21*-H95 were grown in 34/30°C, with 12 h light/12 h dark for 10 days. The middle sections of the 4th leaves were wrapped in aluminum foil to avoid exposure to light and the temperature was changed to 22/18°C. As before, lesions appeared within two or three days on the mutant segregants. After four days the foil was removed. The tissue that had been covered by the foil had virtually no lesions but after two further days at the lower temperature (with continued exposure to light) lesions started developing in these regions also (Figure 4). This experiment was repeated a second time with essentially similar results.

It should be noted that wrapping a portion of the leaf in aluminum foil as well as blocking out light, may have the effect of locally increasing the humidity and possibly also the temperature of the wrapped part of the leaf. So, conceivably, altered temperature and humidity might underlie the failure to form lesions. However, we have observed in other experiments that *Rp1-D21* plants grown in bottles at ~100% humidity display characteristic lesions (data not shown). Therefore it seems that both light and a permissive temperature are required for *Rp1-D21*-associated lesions to form.

### Temperature sensitivity of the defense response associated with *Rp1-D21*

**Figure 5:**
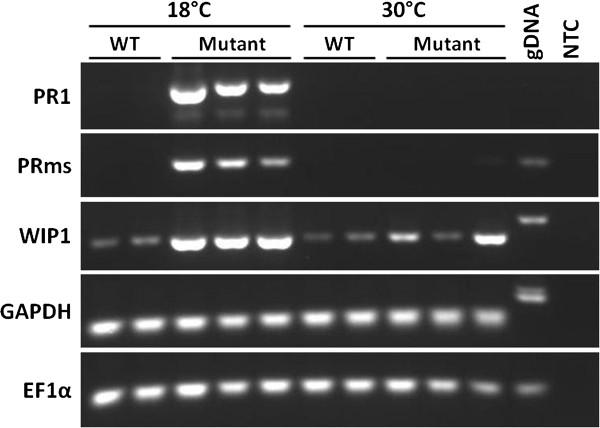
**The expression of three maize genes associated with the defense response *PR1, PRms *and *WIP1 *was measured using semi-quantitative PCR in three mutant two wild type seedlings derived from the cross Tx303 × *Rp1-D21*-H95.***GAPDH* and *EF1α* were included as reference genes. RNA was extracted from the plants grown at a constant 30°C for three weeks, the temperature was then dropped to a constant 18°C for seven days and RNA was extracted from leaves of the same plants.

Apart from the obvious lesion formation phenotype, we have shown previously that *Rp1-D21* expression is associated with other characteristics of the classical HR response including the generation of H_2_O_2_ and superoxide as well as the induction of expression of defense-related genes such as *PR1*, *PR5*, *PRms* and *WIP1*[3]. In order to determine whether the *Rp1-D21* phenotype could also be suppressed at the molecular (as opposed to the phenotypic) level at elevated temperatures, the following experiment was performed: Twelve seeds from a Tx303 × *Rp1-D21*-H95 F1 family were germinated and grown in a growth chamber for three weeks at 30°C. At this point all 12 plants appeared as wild type and leaf samples were taken from all of them. The temperature was then reduced to 18°C and within 48 hours, as expected, lesions were visible on the entire area of six of the plants, these were the segregants carrying the *Rp1-D21* gene while the plants without lesions were their wild type, near-isogenic, siblings. Leaf samples from two wild type and three mutant plants were taken at the four week time-point (i.e. a week after reducing the temperature). Semi-quantitative RT-PCR analysis was performed on RNA extracted from the leaves of these five plants sampled at both the three- and four-week timepoints (i.e. before and after the temperature drop). The results indicated that, after three weeks of continuous growth at 30°C, *PR1* and *PRms* were not detectably expressed while *WIP1* was expressed at relatively low levels in both wild type and mutant segregants. After a week at 18°C, all three of these genes were highly induced relative to their levels at 30°C in mutant but not in wild type plants (Figure 5). This therefore indicates that the defense response (at least as reflected by defense-related gene expression of these specific genes) associated with *Rp1-D21* lesion phenotype is affected by temperature in the same way as the visible HR response.

While this is, to our knowledge the first detailed demonstration of this phenomenon in monocots, examples of temperature sensitive plant disease resistance genes have been long been known, with high temperatures often suppressing function [22]. The tobacco *N* gene, which confers resistance to TMV and the Arabidopsis *RPW8* gene, which confers resistance to powdery mildew are, like *Rp1-D21*, not effective above 30°C [27,28]. Recently the temperature responses of two NB-LRR genes, the *N* gene and the Arabidopsis *SNC1* gene, another NB-LRR gene associated with the defense response [23], were investigated. It was shown that the temperature sensitivity of the defense response they mediated was likely resided in the NB-LRR gene products themselves and not in some component of the downstream signal transduction pathway. Our work here further suggests that while the NBS-LRR gene product may be the primary cause of temperature-sensitivity the interaction of other loci and alleles can modulate this effect. Alcázar and Parker [18] reviewed the interaction of temperature and immune responsiveness in Arabidopsis (the system in which most of this work has been done). They suggest that temperature sensitivity might be a characteristic associated with the molecular assembly of which the NB-LRR gene is a part, but which also includes other proteins such as HSP90. They also suggest that, given the prevalence of this type of relationship, temperature sensitivity may have evolved as an adaptive strategy to ‘tune’ the defense response to the local environmental conditions to balance the advantages of a robust defense response (disease resistance) with the disadvantages such as reduced growth and reproductive potential [29,30].

The basal defense response in Arabidopsis has been shown to be regulated in a circadian manner [31]. The expression levels of several genes important for resistance to *Hyaloperonospora arabidopsidis* and for basal resistance were shown to peak around dawn, the time of peak spore dissemination. In this way, it was hypothesized, the plant was able to synchronize its maximal defense responsiveness to the period of maximal need. Of course dawn is also the coldest period of the day and so perhaps the temperature sensitivity of NB-LRR resistance complexes is another way of fine tuning this system. Similarly, light dependence of the HR mediated by other R-genes has been observed [24,32]. In some cases, this may reflect a relationship between HR, photosynthesis and the oxidative balance in the cell [33,34].

We have been using *Rp1-D21* as a reporter for the HR in order to map loci important for controlling natural variation in the maize defense response [3,14,15]. This work is being performed largely in field experiments in North Carolina and Indiana. The results reported here suggest that environmental temperature will be an important factor in determining how severe the *Rp1-D21* phenotype is in the field and, more importantly, that different temperatures may differentially affect different genotypes. This is not necessarily surprising. Genotype by environment interactions are observed in the vast majority of quantitative trait mapping studies, emphasizing the need for conducting studies over multiple environments.

## Conclusions

We have demonstrated here that:

1. The *Rp1-D21* associated lesion phenotype is temperature-sensitive at both a morphological and a molecular level.

2. *Rp1-D21* temperature-sensitivity is observed in all genetic backgrounds studied, including backgrounds that both strongly enhance and strongly suppress the phenotype.

3. However, there is a significant interaction between temperature and genotype. In other words, the response of the *Rp1-D21* phenotype to changes in temperature is, in part, dependent on genetic background.

4. The effect of genotype on temperature sensitivity can be objectively quantified by examining the kinetics of lesion formation after temperature change.

5. The *Rp1-D21* phenotype can be suppressed at temperatures above 30°C in all genetic backgrounds studied at both the gross phenotypic and the molecular level.

6. The *Rp1-D21* phenotype can be suppressed and activated in a reversible manner by altering the external temperature.

7. Light exposure is also required for the *Rp1-D21* lesion phenotype.

As noted above, temperature effects on the R-gene mediated defense response may be a general phenomenon in plants [18,23,35]. However this is the first time that the interaction of this effect with genetic background has been demonstrated. This implies that other genetic factors other than the *R*-gene itself are responsible for mediating the effect of temperature. Using the *Rp1-D21* system and the MAGIC [14,16,17] technique we will now be able to examine the loci and genes underlying this response. Furthermore, the precise ability to turn the defense response on and off in a massive and synchronous way provides new resources with which to characterize the molecular and physiological events associated the maize defense response.

## Methods

### Plant materials

The *Rp1-D21*-H95 line was generated as described previously [3]. Briefly, the *Rp1-D21* variant was crossed to the maize inbred line H95, and subsequently backcrossed to the H95 parent four times, while selecting for the HR phenotype indicated by the spontaneous formation of cell death lesions. Since *Rp1-D21* homozygotes in the H95 background are often unable to set seed, this stock is maintained in heterozygous condition by repeatedly crossing it as a male to the H95 inbred. A previous study showed that the *Rp1-D21*-H95 line is 97% identical genetically to H95 with 121.5 Mbp of the genome heterozygous for the recurrent and donor parent alleles out of a total genome size of 2045 Mbp [14]. The *Rp1-D21*- B73 line was produced in the same way as *Rp1-D21*-H95, just substituting B73 for H95.

The *Rp1-D21*-H95 line was crossed to 13 diverse lines. All of these lines and B73 are among the 26 parents of the maize Nested Association Mapping population [26]. F_1_ progenies segregated 1:1 mutant:wild type. Apart from the segregation of the *Rp1-D2*1 gene, these progenies were essentially isogenic.

### Growth conditions

Plants were grown in the phytotron greenhouse facility at North Carolina State University with fixed temperatures of 22/18°C, 26/22°C, and 30/26°C higher/ lower for 12 hours each, respectively. For simplicity these greenhouses (GH) were referred to as LowGH, MidGH, and HiGH, respectively. In each year the experiment was laid out in randomized complete block design with two replicates per year per temperature condition. Within each replicate three wild type and three mutant plants were measured per genotype in 2009 and two plants per genotype in 2010. Each replicate was arranged as a randomized complete block. The experiment was performed in fall 2009 and fall 2010. No artificial lighting was used.

In each GH seeds were planted on a plastic germination tray. As soon as it was possible to distinguish wild types from mutant counterparts, seedlings were transplanted to a six inch pot with standard soil mix. Throughout the study period seedlings were watered two times a day on regular basis. Changes in growth and lesion development were photographed and documented.

For all the growth chamber experiments light level were maintained at approximately 325 micromoles/m/s using cool white fluorescent lights. Growth chamber experiments were performed to determine whether constant temperatures above 30°C could abrogate the expression of *Rp1-D21* entirely and to determine whether the phenotype was reversible. Four F_1_ families: Tx303 × *Rp1-D21*-H95, M162W × *Rp1-D21*-H95, and M37W × *Rp1-D21*-H95, B97 × *Rp1-D21*-H95 were used for this experiment. In the first three of these F_1_ families, the *Rp1-D21* phenotype is relatively enhanced; in the last it is relatively suppressed. The *Rp1-D21*-H95 and *Rp1-D21*-B73 lines were also included as standard checks for comparison. Eight plants were grown for each of the crosses in two growth chambers. Both growth chambers were initially calibrated at low temperature of 30°C and high temperature of 34°C. Illumation was fixed at 12 h dark and 12 h light and seedlings were regularly watered twice a day. In one growth chamber, after three weeks (corresponding to the four leaf stage), the temperature was changed to a low temperature of 18°C and high temperature of 22°C. After four weeks the temperature in this growth chamber was shifted back to 30/34°C. Temperature shifts took about 20 minutes to complete in every case. Light duration remained unchanged.

For the experiment to measure the kinetics of *Rp1-D21*-associated lesion formation, F*1* crosses of *Rp1-D21-*H95 crossed to A632, B73, Mo17 and Tx303 were used. Twenty plants from each of the four F_1_ families cross were grown in pots (2 plants per pot) in a growth chamber under a daily photoperiod of 12 h. Initially the temperature of the growth chamber was maintained at a constant temperature of 30°C, but after 8-days of growth (V2 stage) the temperature was dropped to 26°C. Plants expressing HR lesions were counted twice every day for the next 9 days.

### Phenotypic data collection

Plants were evaluated for a lesion severity phenotype on a 0–10 scale with 0 being no symptoms and 10 being dead. Wild type to mutant height ratio was also measured. This was simply the average height of the mutant plants divided by the average height of the wild type plants [3,14]. Lesion severity was scored twice in both fall 2009 and fall 2010.

### Data analysis

ANOVA was performed using SAS version 9.2 (SAS Institute, 2002–2008). SAS Version 9.2 PROC CORR (SAS Institute, 2002–2008) was used to estimate the Pearson correlation coefficients. Heritability estimates were calculated using PROC MIXED procedure of SAS, as described previously [36].

### Semi-quantitative RT-PCR

Total RNA was extracted from maize leaf tissue using Trizol (Life Technologies, Carlsbad, CA) according to manufacturer’s instructions. RNA concentration, quality and integrity were monitored by the NanoDrop and agarose gel electrophoresis. For cDNA synthesis, 1μg of total RNA was reverse transcribed using M-MLV (Life Technologies, Carlsbad, CA) following standard protocols. Briefly, total RNA was mixed with 1μl of Oligo (dT)20 primers (Life Technologies, Carlsbad, CA), heat denatured at 65°C for 5 min and chilled on ice for 2 min. To each reaction 4 μl of 5× First Strand buffer, 2 μl of 0.1 M DTT, and 1μl RNaseOUT (Life Technologies, Carlsbad, CA) was added. The reaction was incubated at 37°C for 2 min and then 1 μl of M-MLV was added, to a final volume of 20 μl. cDNA synthesis was performed at 37°C for 2 hours, followed by a 15 min incubation at 75°C for enzyme inactivation. The cDNA reaction was then diluted 5× using water.

The cDNAs were used for semi- quantitative measurements of gene expression using PCR. Primers PR1-F (5′-AGGCTCGCGTGCCTCCTAGCTCTGG-3′) and PR1-R (5′-GGAGTCGCGCCACACCACCTGCGTG-3′), PRms-F (5′-ACCTGGAGCACGAAGCTGCAG-3′) and PRms-R (5′-GCAGCCGATGCTTGTAGTGGC-3′), WIP1-F (5′-TGCTGATCCTGTGCCTCCAG-3′) and WIP1-R (5′-CTCTCTGATCTAGCACTTGGGG-3′) were utilized to amplify the maize defense genes *PR*1, *PRms* and *WIP1*, respectively [3]. The maize genes *EF1α* and *GAPDH2* were used as reference and amplified using primers EF1a-F1 (5’- ATCTGAAGCGTGGGTATGTG-3’) and EF1a-R1 (5’- GCATAGCCATTGCCAATCTG-3’) and GAPDH2-F2 (5’- GACTTCCTTGGTGACAGCAGG-3’) and GAPDH2-R2 (5’- CTGTAGCCCCACTCGTTGTC-3’), respectively. The *EF1a* and *GAPDH2* primers were from Hachez et al. [37] and slightly modified as needed to minimize dimer formation. Amplification conditions consisted of 32 cycles of 94° for 30 sec, 57° for 30 sec, and 72° for 30 sec, using 250 uM of each primer and 1–4 uL of the 5× diluted cDNA reaction. All primers were obtained from Sigma-Aldrich (St. Louis, MO). Amplification products were analyzed by agarose gel electrophoresis. Genomic DNA contamination was monitored by the size of the amplification product for the *GAPDH* gene using B73 genomic DNA as positive control for the PCR.

## Competing interests

The authors declare that they have no competing interests.

## Authors’ contributions

AN, GJ, PBK planned the project. AN, GFW performed experiments examining the interactions between genetic background and temperature. LB planned and performed the RT-PCR experiments. CMK planned and performed the experiments for the objective quantification of the background effect on temperature. GFW, VC planned and performed the experiments on the effect of light. AN, PBK analyzed the data. AN, PBK wrote the manuscript. AN, GJ, PBK, GFW edited the manuscript. All authors read and approved the final manuscript.

## Supplementary Material

Additional file 1: Figure S1 The scoring scale used for visual scoring of the lesions. Leaves scored from 1–8 are shown. An entirely dead leaf would score a “10” whereas a “9” would be a leaf with just a few patches, about 5%, of living tissue. Click here for file

Additional file 2: Figure S2 Plants in 13 different backgrounds were grown for four weeks at 22/18°C, 12 hr light/dark and then shifted to 34/30°C 12 hr light/dark. At this point at which the 5th leaves were partially emerged from the whorl. Pictures were taken from 5th leaves five days after temperature shift. In every case the genotype of the plant is an F_1_ cross between the line indicated and *Rp1-D21*-H95. The red line indicates the point up to which the 5th leaf had emerged at the time of the temperature shift. While only one plant is pictured for each background, at least 3 mutant F_1_ plants were observed in each background and the results were essentially similar in each case. Click here for file
