# Supplementary figures and images for: Characterization of temperature and light effects on the defense response phenotypes associated with the maize Rp1-D21 autoactive resistance gene
Source: BMC Plant Biol. 2013 Jul 26;13:106. doi: 10.1186/1471-2229-13-106 (PMC3733612; doi:10.1186/1471-2229-13-106)

## Slide 1
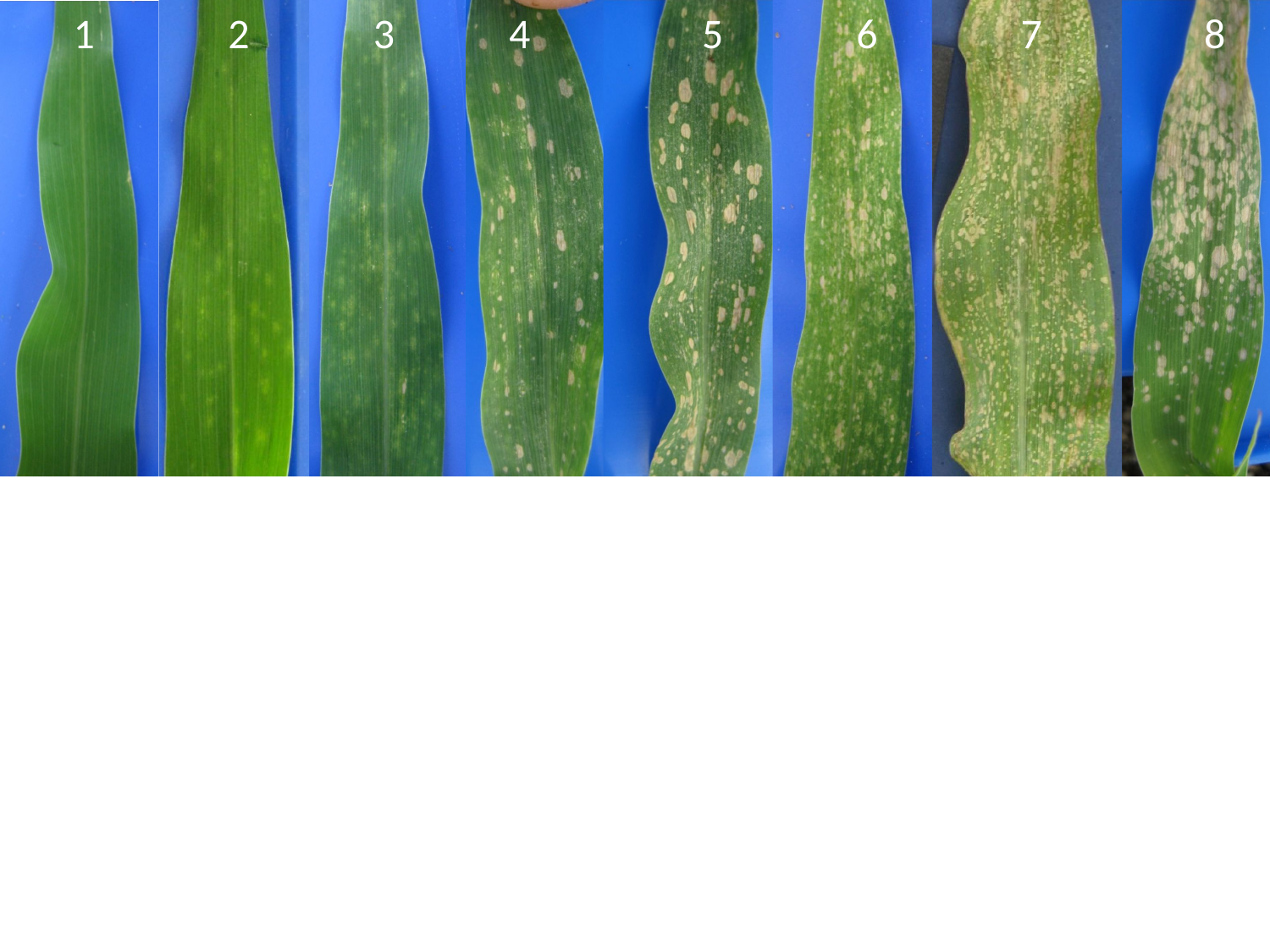

1 2 3 4 5 6 7 8
#

Supplement: Additional file 1: Figure S1 — The scoring scale used for visual scoring of the lesions. Leaves scored from 1–8 are shown. An entirely dead leaf would score a “10” whereas a “9” would be a leaf with just a few patches, about 5%, of living tissue. [file 1471-2229-13-106-S1.pptx]

## Slide 1
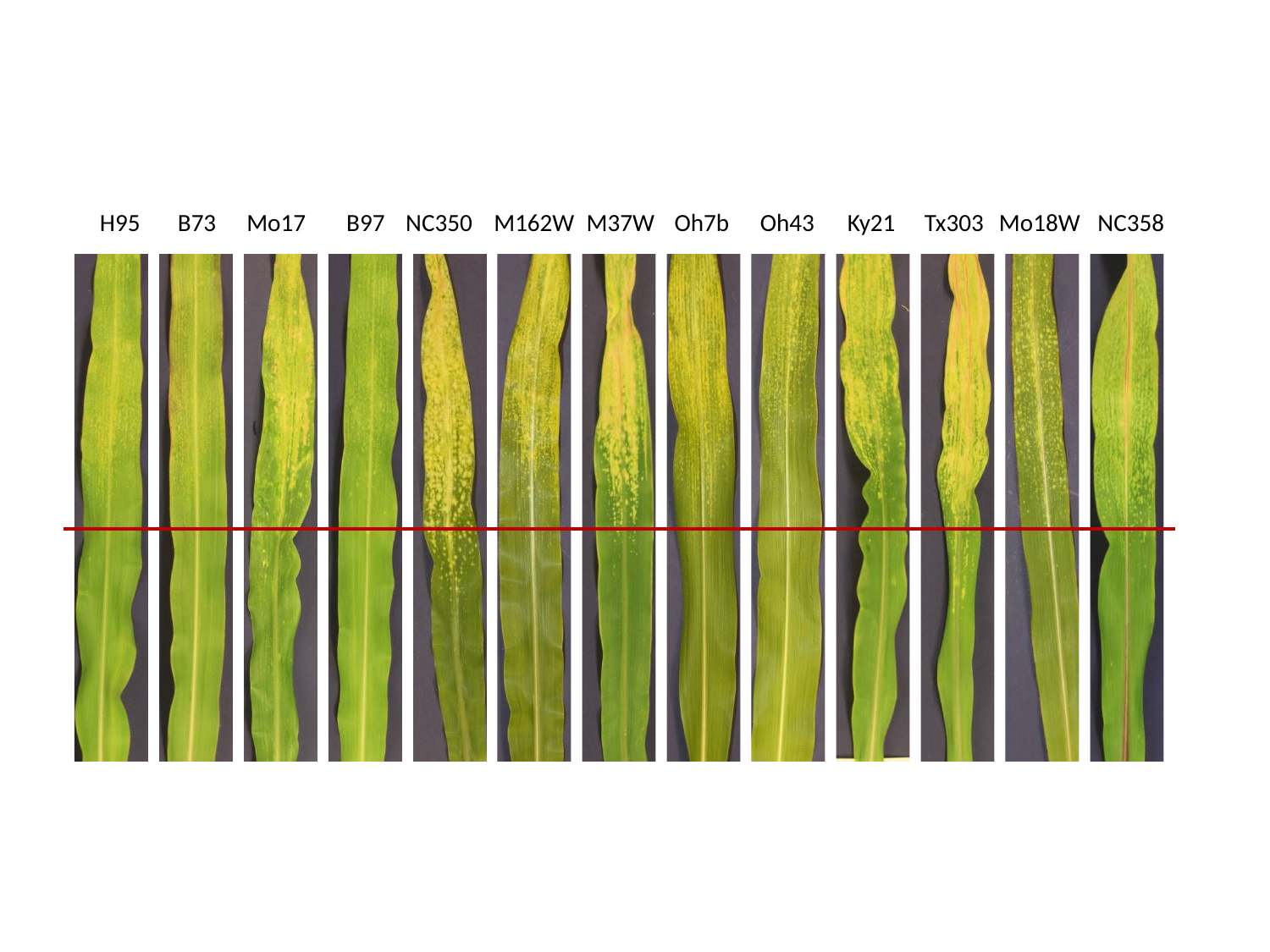

H95
B73
Mo17
B97
NC350
M162W
M37W
Oh7b
Oh43
Ky21
Tx303
Mo18W
NC358

Supplement: Additional file 2: Figure S2 — Plants in 13 different backgrounds were grown for four weeks at 22/18°C, 12 hr light/dark and then shifted to 34/30°C 12 hr light/dark. At this point at which the 5th leaves were partially emerged from the whorl. Pictures were taken from 5th leaves five days after temperature shift. In every case the genotype of the plant is an F1 cross between the line indicated and Rp1-D21-H95. The red line indicates the point up to which the 5th leaf had emerged at the time of the temperature shift. While only one plant is pictured for each background, at least 3 mutant F1 plants were observed in each background and the results were essentially similar in each case. [file 1471-2229-13-106-S2.pptx]
